# In utero exposure to ritodrine during pregnancy and risk of autism in their offspring until 8 years of age

**DOI:** 10.1038/s41598-020-80904-y

**Published:** 2021-01-13

**Authors:** Jungsoo Chae, Geum Joon Cho, Min-Jeong Oh, KeonVin Park, Sung Won Han, Suk-Joo Choi, Soo-young Oh, Cheong-Rae Roh

**Affiliations:** 1 Department of Obstetrics and Gynecology, Samsung Medical Center, Sungkyunkwan University School of Medicine, 81 Irwon-ro, Gangnam-gu, Seoul, 06351 Republic of Korea; 2 Department of Obstetrics and Gynecology, Korea University Guro Hospital, Korea University College of Medicine, 148 Gurodong-ro, Guro-gu, Seoul, 08308 Republic of Korea; 3 Department of Statistics, Seoul National University, Seoul, Republic of Korea; 4 School of Industrial Management Engineering, Korea University, Seoul, Republic of Korea

**Keywords:** Epidemiology, Paediatric research

## Abstract

Beta-2 adrenergic receptor (B2AR) agonists, used as asthma treatments and tocolytics during pregnancy, have recently been reported to be associated with autism in their offspring. However, the particular link between autism and ritodrine, a common type of B2AR agonist used solely as tocolytics, has never been substantiated with any nationwide database. Thus, we aimed to examine the association between in utero exposure of ritodrine and the risk of autism in their offspring using a national database. This population-based cohort study was conducted by merging the Korea National Health Insurance claims database and National Health Screening Program for Infants and Children database. These databases included all women who had delivered singleton between January 2007 and December 2008 in Korea. Out of the total 770,016 mothers, 30,959 (4.02%) were exposed to ritodrine during pregnancy, and 5583 (0.73%) of their children were identified as having autism, defined until 8 years of age. According to our analysis, the overall cumulative incidence of autism up to 8 years was 1.37% in ritodrine exposure group and 0.70% in ritodrine non-exposure group (*p* < 0.05, log-rank test). By Cox proportional hazard analysis, use of ritodrine in preterm birth was associated with significantly higher hazard of autism [adjusted hazard ratio: 1.23, 95% CI 1.04–1.47], after adjusting for confounding variables including maternal age, parity, cesarean section, preterm labor, steroid use, birth weight, gender, and preeclampsia. Thus, in utero exposure to ritodrine was associated with an increased risk of autism in their offspring.

## Introduction

Preterm birth, a leading cause of neonatal mortality and morbidity, is one of the primary concerns among many obstetricians and pediatricians^1^. The prevalence of preterm birth has been reported as 7.5% in the developed countries and 12.5% in developing countries^2^. Overall, it occurred in 11.1% births worldwide in 2010^3^ and in nearly 7% of all births in Korea according to Korean National Statistics in 2016^4^. Nowadays, a number of medical strategies for prevention and management of impending preterm births are implemented in obstetric practices, including tocolytics, progesterone, and antibiotics. However, there is a significant lack of data with regards to the long term effects of such medicines on childhood outcomes, when used for mothers during their antenatal period^5^.

As a preventative measure for preterm birth, tocolytics therapy is usually recommended to secure time before the subsequent antenatal corticosteroid treatment. This therapy is known to be effective in delaying gestational age at delivery up to 48 h or 7 days, but its ability to improve neonatal outcomes has not been confirmed^6,7^. Such tocolytics include beta-2 adrenergic receptor (B2AR) agonist, calcium channel blocker, oxytocin antagonist, and magnesium sulfate, all prescribed for preventing preterm labor^8^.

Among these tocolytics, a long-term usage of B2AR agonists has been discouraged by many recent guidelines due to its potential side effects^8–14^. For instance, supply of ritodrine was voluntarily withdrawn from the United States market by the manufacturer in 1998 due to severe maternal side effects such as cardiovascular complication and death^15,16^. In addition, in 2011, the United States Food and Drug Administration restricted the prolonged use of terbutaline as tocolytics^10^. In 2013, European Medicines Agency also restricted the use of oral and suppository forms of short acting beta-agonists due to cardiovascular side effects^11^, and consequently, the National Institute for Health and Care Excellence guideline in 2015 prohibited the use of beta-mimetics for tocolytic purposes^12^.

It is well known that B2AR agonists may have maternal side effects including pulmonary edema, palpitation, and hypokalemia, along with various fetal-neonatal side effects such as fetal tachycardia^17^ and neonatal hypoglycemia^18^. In addition to these short-term side effects, deleterious behavioral effects in offspring after in utero exposure to various B2AR agonists in mothers have been recently reported. A study by Connors et al. in 2005 showed that prenatal exposure of terbutaline for 2 weeks or longer was associated with increased concordance rate for autism in dizygotic twins^19^. Another study from the Unites States in 2011 suggested that exposure of terbutaline during the third trimester may be associated with an increased risk of autism^20^. Later studies using Demark population registers also demonstrated that in utero exposure of B2AR agonists was associated with increased risk of autism and attention-deficit/hyperactivity disorders-risk^21,22^.

However, most studies above defined B2AR agonist exposure during pregnancy based on the outpatient prescriptions, and therefore included multiple types of B2AR agonists. In addition, since B2AR agonists are also commonly used in treatment of asthma, the association between in utero exposure of B2AR agonists and autism in their offspring demonstrated by the studies above may not specifically apply to situations in which B2AR agonists are used for tocolytic purpose only. Meanwhile, ritodrine is used solely as tocolytics unlike many other B2AR agonists and is still one of the most common tocolytics in clinical practice in many countries including Korea, Canada, Japan^23–25^. Therefore, there is an urgent need to examine the link between in utero exposure of ritodrine and autism in their offspring using a nationwide birth database. Using a comprehensive database from the Korea National Health Insurance (KNHI) and National Health Screening Program for Infants and Children (NHSP-IC), we identified all women who delivered singleton between January 2007 and December 2008 (*n* = 770,016). Then, based on International Classification of Diseases-10th Revision (ICD-10) codes, we examined the cumulative diagnosis of autism in their offspring until 2015.

## Materials and methods

### Characteristics of the data

This study was conducted by integrating the KNHI claims database and NHSP-IC database. The KNHI program covers 97% of the population; the Medical Aid Program is usually in charge of the remaining 3%. The KNHI claims database contains information on claims for approximately 50 million Koreans. Thus, nearly all information about the varied disease prevalence and treatments can be provided by this centralized database, with the exception of procedures not covered by insurance such as cosmetic surgery.

The KNHI system provides an NHSP-IC for all neonates for seven consecutive health examinations based on age groups (4 to 9 months, 9 to 18 months, 18 to 30 months, 30 to 42 months, 42 to 54 months, 54 to 66 months, and 66 to 80 months). The NHSP-IC consists of two components: a health interview with the parents and a health examination of offspring. NSHP-IC also offers screening for developmental delay, the Korean Ages and Stages Questionnaire (K-ASQ). Children suspected of a developmental delay on the K-ASQ questionnaire are transferred to specialized clinic. The study population was only composed of neonates who underwent NHSP-IC therefore these neonates were included in screening for developmental disorders.

### Study population

**Figure 1:**
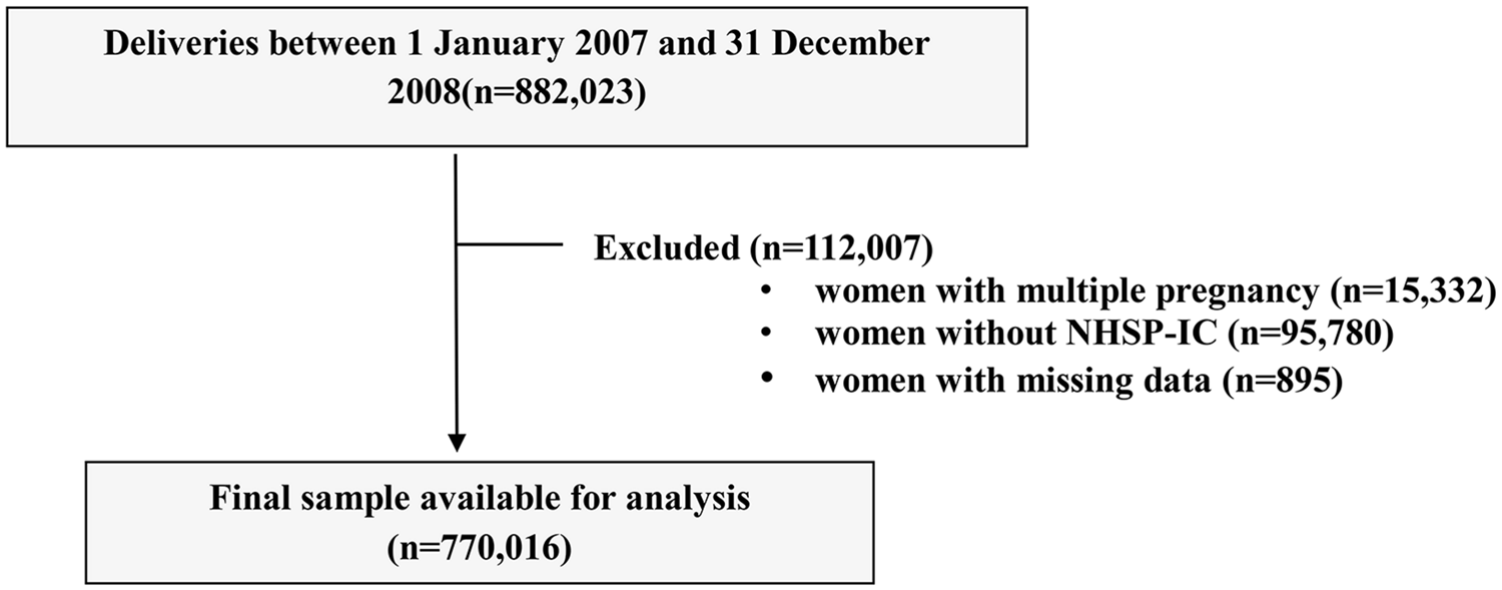
Study population.

A flowchart of patient enrollment is shown in Fig. 1. Using the KNHI claims database, we identified all women who delivered singleton between January 1, 2007 and December 31, 2008. Women were excluded from the analysis if their offspring did not undergo at least one of the seven consecutive NHSP-IC or had missing data to identify the pregnancy outcomes such as preterm birth and birthweight. The Institutional Review Board of Korea University Medical Center (IRB No. 2019GR0310) reviewed and approved this study. The IRB of Korea University Medical Center waived the need for informed consent due to de-identification of the KNHI database. The present study was performed in accordance to the latest version of the Declaration of Helsinki.

### Outcome ascertainment

Using the KNHI claims database, autism was identified via primary or secondary diagnosis based on ICD-10 codes. Offspring were classified as having autism if they were diagnosed with autism (ICD-10 code F84.X) from birth to December 31, 2015.

### Covariates

Maternal use of ritodrine during index pregnancy was identified using the KNHI claims database. Based on ICD-10 code, information about pregnancy outcomes including parity, cesarean delivery, preterm labor, steroid use, and preeclampsia were evaluated using the KNHI claims database. Information on neonatal outcomes including preterm birth, neonatal birth weight, and gender data were collected from the NHSP-IC database. Preterm birth was defined as gestational age < 37 weeks, low birth weight was defined as birth weight < 2500 g, and large for gestational age was defined as birth weight > 4000 g.

### Statistical analysis

Differences in continuous and categorical variables were analyzed using the t-test and Chi-square test, respectively. The cumulative incidence of autism was estimated using the Kaplan–Meier method and compared using the log-rank test. Cox proportional hazards models were used to estimate the adjusted hazard ratios (HRs) and 95% confidence intervals (CIs) for the development of autism. A *p* value of < 0.05 was considered statistically significant. Statistical analyses were performed using SAS for Windows, version 9.4 (SAS Inc., Cary, NC, USA).

## Results

A total of 882,023 women delivered in South Korea from January 2007 to December 2008. Among these women, 112,007 were excluded from the study for the following reasons. As demonstrated in Fig. 1, 15,332 women had multiple pregnancies and 95,780 women did not register their offspring in NHSP-IC. Data for covariates used in this study were missing in 895 women, who were also excluded. Out of the resulting 770,016 children, 5583 (0.73%) were diagnosed with autism between birth and 8 years of age.

Among the 770,016 mothers, 30,959 (4.02%) were exposed to ritodrine during pregnancy.

Table 1 summarizes the clinical characteristics of mothers with and without ritodrine exposure. Compared to those without ritodrine exposure (controls), mothers with ritodrine exposure were younger, more multiparous, and had more cesarean delivery. In addition, they were more likely to experience preterm birth, preterm labor, steroid use, and low birth weight. In the ritodrine exposure group, the percentage of gender-male and preeclampsia were also higher.

**Table 1:** Clinical characteristics of the mothers with and without ritodrine exposure.

	Controls (*N* = 739,057)	Ritodrine exposure (*N* = 30,959)	*p*-value
Maternal age (year)	30.2 ± 3.8	30.0 ± 3.9	< 0.01
Maternal age (n, %)			< 0.01
≤ 25	68,967 (9.3)	3418 (11.0)	
26–30	343,288 (46.5)	14,506 (46.9)	
31–35	264,627 (35.8)	10,385 (33.5)	
36–40	57,483 (7.8)	2424 (7.8)	
≥ 41	4,692 (0.6)	226 (0.7)	
Multiparity (n, %)	395,488 (53.5)	18,648 (60.2)	< 0.01
Cesarean delivery (n, %)	260,564 (35.3)	12,227 (39.5)	< 0.01
Preterm birth (n, %)	14,493 (2.0)	5644 (18.2)	< 0.01
Preterm labor (n, %)	5392 (0.7)	2,833 (9.2)	< 0.01
Steroid use (n, %)	15.190 (2.1)	3336 (10.8)	< 0.01
Birth weight (kg)	3.23 ± 0.47	2.95 ± 0.65	< 0.01
Low birth weight (n, %)	23,489 (3.2)	5066 (16.4)	< 0.01
Large for gestational age (n, %)	32,120 (4.4)	739 (2.4)	< 0.01
Gender-male (n, %)	379,973(51.4)	17,038 (55.0)	< 0.01
Preeclampsia (n, %)	24,564 (3.3)	1,829 (5.9)	< 0.01

Data presented is n (%) or mean (standard deviation).

**Figure 2:**
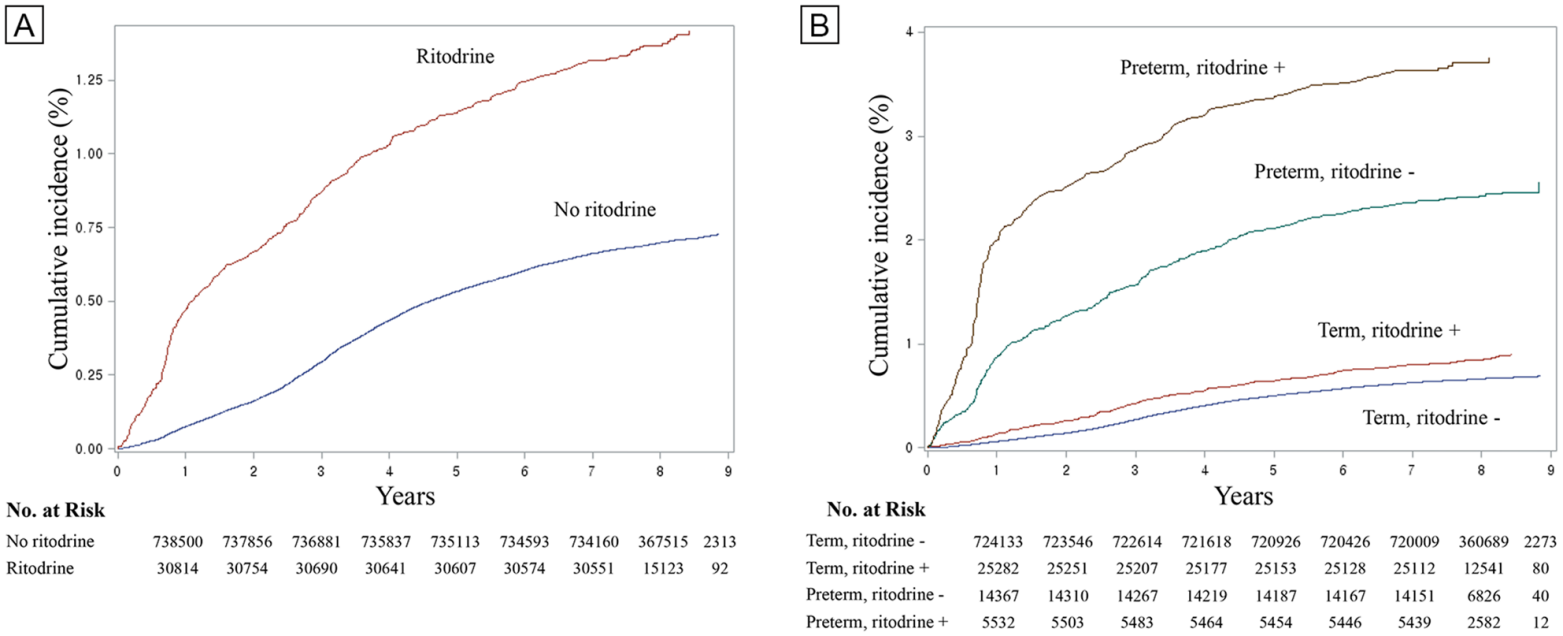
(**A**) Cumulative incidence of autism up to 8 years of age depending on ritodrine exposure. (**B**) Subgroup analysis; cumulative incidence of autism up to 8 years of age by preterm and term birth on ritodrine use.

Figure 2A demonstrates the cumulative incidence of autism up to 8 years of age depending on ritodrine exposure during pregnancy. Overall, cumulative incidence of autism up to 8 years was 1.37% in ritodrine exposure group and 0.70% in ritodrine non-exposure group (*p* < 0.05, log-rank test).

Table 2 shows the HRs of autism after maternal ritodrine exposure, calculated through Cox proportional hazard analysis. The unadjusted HR was 1.98 (95% CI, 1.79–2.19). The hazard of autism remained significantly higher in ritodrine exposure group with an adjusted HR of 1.12 (95% CI 1.07–1.32), even after taking account of confounding variables such as maternal age, parity, cesarean section, preterm birth, preterm labor, steroid use, low birth weight, large for gestational age, male gender, and preeclampsia. Variables including maternal age, cesarean delivery, preterm birth, preterm labor, low birth weight, male gender and preeclampia were associated with significantly higher adjusted HRs for autism. Otherwise, multiparity and steroid use were associated with lower adjusted HRs for autism.

**Table 2:** Hazard ratios (HRs) of autism according to maternal ritodrine exposure and other risk factors.

	Unadjusted HR (95% CI)	Adjusted HR (95% CI)
Ritodrine exposure	1.98 (1.79, 2.19)	1.12 (1.07, 1.32)
Maternal age (years)		
≤ 25	1.02 (0.93, 1.13)	0.98 (0.89, 1.08)
26–30	1	1
31–35	1.12 (1.05, 1.19)	1.21 (1.14, 1.29)
36–40	1.39 (1.27, 1.53)	1.47 (1.34 1.62)
≥ 41	1.95 (1.52, 2.50)	1.92 (1.50, 2.47)
Multiparity	0.72 (0.68, 0.76)	0.66 (0.62, 0.70)
Cesarean delivery	1.30 (1.23, 1.37)	1.15 (1.09, 1.21)
Preterm birth	4.24 (3.88, 4.62)	2.80 (2.39, 3.28)
Preterm labor	3.37 (2.92, 3.89)	1.35 (1.15, 1.58)
Steroid use	2.05 (1.81, 2.31)	0.55 (0.47, 0.64)
Low birth weight	3.73 (3.44, 4.03)	2.19 (1.95, 2.46)
Large for gestational age	0.97 (0.85, 1.10)	0.94 (0.83, 1.08)
Gender—male	2.53 (2.39, 2 67)	2.53 (2.39, 2.69)
Preeclampsia	1.66 (1.48, 1.86)	1.13 (1.00, 1.27)

Hazard ratios were adjusted for variables in table. HR, hazard ratio.

Since preterm birth itself is a significant risk factor for autism in their offspring, we also performed a subgroup analysis by dividing preterm and term delivery groups.

Figure 2B demonstrates the same cumulative incidence, but using the subgroup analysis of preterm and term birth. The cumulative incidence of autism up to 8 years was highest in preterm birth with ritodrine exposure group (3.70%), followed by preterm birth with ritodrine non-exposure group (2.43%), term birth with ritodrine (0.85%), and term birth without ritodrine (0.66%) (*p* < 0.05, log-rank test).

Table 3 shows the subgroup results using preterm birth without ritodrine as the reference group. Here, the hazard of autism significantly increased in preterm birth with ritodrine group (adjusted HR 1.23, 95% CI: 1.04–1.47) after adjusting for confounders.

**Table 3:** Hazard ratios (HRs) of autism in term and preterm birth; subgroup analysis of autism with respect to preterm birth without ritodrine group.

	Ritodrine exposure	Unadjusted HR (95% CI)	Adjusted HR (95% CI)
**Term birth**	−	0.27 (0.24, 0.30)	0.36 (0.31, 0.43)
	+	0.35 (0.29, 0.41)	0.42 (0.34, 0.52)
**Preterm birth**	−	1	1
	+	1.54 (1.30, 1.83)	1.23 (1.04, 1.47)

Hazard ratios were adjusted for maternal age, parity, cesarean section, preterm labor, steroid use, low birthweight, large for gestational age, male gender, and preeclampsia.

Table 4 shows the subgroup results using no preterm labor without ritodrine as the reference group. The hazard of autism diagnosed increased in no preterm labor with ritodrine (adjusted HR: 1.34, 95% CI: 1.10–1.50), preterm labor without ritodrine (adjusted HR: 1.58, 95% CI: 1.29–1.94), and preterm labor with ritodrine (adjusted HR: 2.44, 95% CI: 1.97–3.02), after adjusting for confounders.

**Table 4:** Hazard ratios (HRs) of autism for women with and without preterm labor; subgroup analysis of autism with respect to no preterm labor without ritodrine group.

	Ritodrine exposure	Unadjusted HR (95% CI)	Adjusted HR (95% CI)
**Preterm labor −**	−	1	1
	+	1.73 (1.55, 1.93)	1.34 (1.10, 1.50)
**Preterm labor +**	−	2.80 (2.30, 3.40)	1.58 (1.29, 1.94)
	+	4.73 (3.85, 5.81)	2.44 (1.97, 3.02)

Hazard ratios were adjusted for maternal age, parity, cesarean section, preterm delivery, steroid use, low birthweight, large for gestational age, male gender, and preeclampsia.

## Discussion

Using a national database that encompasses nearly all infants born in Korea between 2007 and 2008, this study demonstrates that maternal in utero exposure of ritodrine during pregnancy is associated with an increased hazard of autism in their offspring until 8 years of age. By Cox proportional hazard analysis, the use of ritodrine during pregnancy is generally associated with higher hazard of autism, even after adjusting confounders such as maternal age, neonatal birth weight, preterm birth, preterm labor and steroid use (HR 1.12, 95% CI:1.1.07–1.32). Moreover, the use of ritodrine in preterm birth was associated with significantly higher hazard of autism [HR 1.23, 95% CI 1.05–1.47], after adjusting for confounding variables.

Autism spectrum disorder (ASD) describes a wide range of symptoms, including difficulty with social interaction, lack of communication skills, and unusually repetitive behavior^26^. The global prevalence of ASD was reported as 0.62% in a review published in 2012^27^. In addition, its prevalence has increased in the United States from 0.67% in 2000 to 1.46% in 2012, according to the Autism and Developmental Disabilities Monitoring Network (ADDM) report^28^. A recent study using the total population of South Korean children aged 7–12 years revealed remarkably high ASD prevalence as 2.64%, accounting for 1/38 of the total sample^29^.

The reason for such increase in ASD is not entirely clear. Environmental effects such as use of medicines during fetal development as well as genetic factors are all suggested as a possible etiology of ASD. A number of studies have recently proposed a link between ASD and exposure to certain medicines, suggesting the importance of intrauterine environment in long-term fetal neurodevelopmental outcomes. For example, use of antidepressants during the second and/or third trimester was associated with an ASD risk (adjusted HR 1.87, 95% CI: 1.15–3.04)^30,31^. In addition, antiepileptic drugs such as valproate were reported to have seven times higher prevalence of ASD when exposed during pregnancy^32^. Recently, it was suggested that paracetamol (acetaminophen) may increase the risk of autism as well as hyperkinetic symptoms (HR 1.51, 95% CI: 1.19–1.92)^33^. Besides these medicines, many others introduced during pregnancy were also associated with increased risk of autism^31^.

Aside from these medicines, ritodrine, a particular type of B2AR agonist, was chosen for our analysis. Our study reveals an increased autism rate (HR 1.12, 95% CI: 1.07–1.32) with prenatal exposure of ritodrine, a result similar to a case–control study in 2016 based on Denmark population-based register. This previous study, which included 5200 cases with ASD and 52,000 controls without ASD, showed the increased odds of ASD (OR 1.3, 95% CI: 1.1–1.5) when exposed to B2AR agonists during pregnancy^21^. However, as this study did not specify the exact purpose of B2AR agonist prescriptions, it seems likely that these agonists were rather prescribed for the treatment of asthma, given that such B2ARs were chosen based on the outpatient prescriptions. Compared to these previous studies^19–22^, the difference in our study is that only the intravenous ritodrine, used only for tocolytic purposes and independently coded by the KNHI database, was included. Moreover, this is the largest cohort study so far, analyzing the association between B2AR and ASD risk using a nationwide database, collected from the Korea National Health Insurance Claims Database and Korea National Health Screening Examination for 2008–2015.

An insight into the mechanism behind ritodrine leading to ASD can be gained through several previous studies. Certain animal studies using rats have reported that an exposure to B2AR agonists during fetal period acts as a neurobehavioral teratogen for fetus and thus impairs the development of peripheral non-adrenergic projections^34–37^. In fetal catecholamine system, norepinephrine binds to B2AR on the fetal rat CNS cell surface, activating adenylyl cyclase through G protein. This produces cyclic adenosine monophosphate and protein kinase A, which results in an activation or inhibition of mitogen-activated protein kinase pathways, ultimately leading to CNS cell growth, differentiation, and apoptosis^38^. Therefore, an overstimulation of B2AR by its ligand, such as terbutaline and ritodrine in immature rat fetal CNS cells, results in a disruption of synaptogenesis particularly in cerebellum^36,39^ and peripheral synaptic projections^35^. Such disruptions in neuro-inflammation patterns and structures are similar to the postmortem findings of children and adults with ASD^36^. Ritodrine has also been reported to pass through the placenta, according to animal experiments using pregnant baboon^40^ and a perfusion study of humans^41^.

Despite such findings, however, there exist several limitations to our study. First, the diagnosis of ASD in this study was based on ICD-10 codes from insurance claims data, not on Diagnostic and Statistical Manual of Mental Disorders (DSM-5). Although several epidemiologic studies on ASD used ICD based diagnosis like our study^20^^.^^21^, we acknowledge that the validity of the diagnoses may be limited by either over-diagnosis or under-diagnosis. In addition, although the Korean Ages and Stages Questionnaire (K-ASQ) by NSHP-IC in our country provides to screen for developmental delays, it may not be perfect to exclude the diagnosis of ASD. Regardless of such limitations, however, the accuracy of the diagnostic codes tends to be higher for more severe conditions including ASD^42^, according to the health insurance system in Korea. Furthermore, the ASD cases in this study were diagnosed by physicians including psychiatric specialists, and the diagnosis were strictly reviewed by Health Insurance Review & Assessment Service in Korea.

Second, we could not analyze the dose response relationship between ritodrine and ASD. Instead, we could find two studies assessing the dose of general B2ARs in association with ASD. According to a study by Gidaya et al., longer exposure to B2ARs (defined as > 45 days) was associated with higher risk of ASD than shorter exposure (1–45 days)^21^. Another study by Croen et al. showed that terbutaline exposure for 2 days or more resulted in about fourfold increase in ASD risk (adjusted OR 4.4, 95% CI: 0.8–24.6), despite certain statistically imprecise effect estimates^20^. Particularly, terbutaline exposure for 1–2 days did not increase the risk for ASD (adjusted OR 1.0, 95% CI 0.5–2.0) and exposure ≥ 2 weeks showed further increased risk (adjusted OR 4.7, 95% CI: 0.4–53.3). In addition, in a review by Witter et al., it was suggested that the duration of high-dose exposure to B2AR agonists was likely to be > 2 weeks^43^. Aside from ASD, the relation between dose-dependent risk of intravenous ritodrine and childhood asthma was also recently demonstrated in a Japanese study that used a hospital-based cohort^44^.

Furthermore, it still remains unclear as to which exact phase during pregnancy has the highest risk of autism when exposed to B2AR agonists. According to a study by Croen et al., third trimester exposure was suggested to have the highest risk for autism than the first and second trimester^20^, although it failed to reach statistical significance. In contrast, in a study by Gidaya et al., all trimesters manifested a similarly high increase risk of autism: first trimester (OR: 1.3, 95% CI: 1.1–1.5), second trimester (OR: 1.5, 95% CI: 1.1–1.7), and the third trimester (OR: 1.4, 95% CI: 1.1–1.7)^21^. Considering that fetal brain development process varies on gestational age, it would have been meaningful to compare the effects of ritodrine exposure depending on gestational age. However, we did not have information on gestational age at ritodrine exposure and could not examine trimester-specific effect estimates of ritodrine.

In addition, since we only analyzed clinical variables provided by KNHI and NHSP-IC database, we might have missed other confounding variables affecting the development of ASD. For example, these databases provided neonatal birth weight but not the exact gestational age at delivery, so our multivariable analysis used only neonatal birth weight. However, given the well-known multicollinearity of gestational age at delivery and neonatal birthweight, our analysis can be considered valid. Last limitation of our study is that we could not analyze the other well-known risk factors associated with childhood autism in our study such as paternal age, parental education, and socio-economic status etc., as this rather personal information was not available in our database system.

This study also confirms several other important perinatal risk factors for autism, including maternal age and male gender as previously mentioned. For example, our analysis demonstrated that mothers aged 36–40 showed adjusted HR of 1.47 (95% CI, 1.34–1.62) for autism in their child, compared to those aged 26–30. Such finding is in line with a recent meta-analysis, which showed that adjusted relative risk for autism in the offspring was 1.31 (95% CI, 1.19–1.45) in mother above 35 years old, compared to mothers 25 to 29 years old^45^. Our data also revealed that male gender is significantly associated with autism development with an HR of 2.53 (95% CI, 2.39–2.69), which also agrees with previous studies^46^.

In summary, the importance of our study lies on its use of a national scale data of a large population-based cohort, in proving the association between ritodrine and autism. Considering the possible long-term adverse outcomes of ritodrine, we reaffirm that the use of ritodrine should be limited to a short period of time, only to complete the corticosteroid for fetal lung maturation.
